# Screening and validation of differentially expressed microRNAs and target genes in hypertensive mice induced by cytomegalovirus infection

**DOI:** 10.1042/BSR20202387

**Published:** 2020-12-10

**Authors:** YunZhong Shi, DongMei Xi, XiaoNi Zhang, Zhen Huang, Na Tang, YongMin Liu, LaMei Wang, Yan Tang, Hua Zhong, Fang He

**Affiliations:** 1 Department of Pathophysiology/Key Laboratory of Education Ministry of Xinjiang Endemic and Ethnic Diseases, Medical College of Shihezi University, Shihezi, China; 2 Second Department of Emergency and Critical Care Medicine, The First Affiliated Hospital of Medical College of Shihezi University, Shihezi, China; 3 The Centre of Medical Functional Experiments, Medical College of Shihezi University, Shihezi, China; 4 Department of Geriatrics, The First Affiliated Hospital of Medical College, Shihezi University, Shihezi, China

**Keywords:** Cytomegalovirus infection, essential hypertension, miRNA

## Abstract

**Introduction:** Multiple studies have suggested an association between cytomegalovirus (CMV) infection and essential hypertension (EH). MicroRNAs (miRNAs) play a critical role in the development of EH by regulating the expression of specific target genes. However, little is known about the role of miRNAs in CMV-induced EH. In the present study, we compared the miRNA expression profiles of samples from normal and murine cytomegalovirus (MCMV)-infected C57BL/6 mice using high-throughput sequencing analysis. **Methods:** We collected the thoracic aorta, heart tissues, and peripheral blood from 20 normal mice and 20 MCMV-infected mice. We identified differentially expressed miRNAs in the peripheral blood samples and predicted their target genes using bioinformatics tools. We then experimentally validated them using quantitative reverse transcription polymerase chain reaction (qRT-PCR) and the target genes with double luciferase reporter gene assay. **Results:** We found 118 differentially expressed miRNAs, among which 9 miRNAs were identified as potential MCMV infection-induced hypertension regulators. We then validated the expression of two candidate miRNAs, mmu-miR-1929-3p and mcmv-miR-m01-4-5p, using qRT-PCR. Furthermore, the dual-luciferase reporter gene assay revealed that the 3′-untranslated region (UTR) of endothelin A receptor (*Ednra*) messenger RNA (mRNA) contained a binding site for mmu-miR-1929-3p. Collectively, our data suggest that MCMV infection can raise the blood pressure and reduce mmu-miR-1929-3p expression in C57BL/6 mice. Moreover, we found that mmu-miR-1929-3p targets the 3′-UTR of the *Ednra* mRNA. **Conclusion:** This novel regulatory axis could aid the development of new approaches for the clinical prevention and control of EH.

## Introduction

Hypertension is one of the most common chronic diseases, affecting more than 1 billion people worldwide [1]. Chronically elevated blood pressure is a common cause of chronic renal failure, myocardial infarction, stroke, heart failure, and death [2]. Approximately 90‒95% of all the hypertension cases are classified as essential hypertension (EH) [3,4]. Although the prevention and treatment of hypertension have made great progress in the past decade, the etiology and pathogenesis of hypertension have not been fully elucidated yet. A recent study showed that hypertension is not only related to the interaction between genetic and environmental factors but also with viral infections and non-coding RNAs, such as microRNA (miRNA) [5,6]. Another population association study showed that the human cytomegalovirus (HCMV) and miRNAs encoded by HCMV are correlated to hypertension [7]. HCMV is a member of the cytomegalovirus (CMV) genus, and the prevalence of HCMV infection has been estimated in the 40–100% range [8]. Moreover, a European study has shown an independent correlation between HCMV infection and blood pressure [9]. However, at present, the relationship between HCMV infection and hypertension remains unclear. Some studies suggested that the mechanism of CMV infection-induced hypertension may be correlated to oxidative stress and endothelial dysfunction [10]. Alternatively, HCMV could promote hypertension through developmental and metabolic disorders to vascular smooth muscle cells [11]. Due to the high prevalence of hypertension and widespread HCMV infection, it is particularly important to clarify the role of HCMV in hypertension pathogenesis.

HCMV is a ubiquitous β herpes virus that has the largest known genome among human herpesviruses (230 kb) encoding a variety of factors that modulate the host immune response [12,13]. HCMV infection can cause latent infection and toxic lytic infection, depending on the host’s response to the infection. In latent infection, the virus does not replicate, although the viral genome and proteins remain in the host cells. When the immune system is normal, HCMV infection will not cause any uncomfortable symptoms [14]. However, when the host defenses are impaired—in fetuses, infants, young children, and people with low immunity—the virus immediately gets activated, causing a toxic lytic infection which can lead to extensive damage [15,16]. A recent study has shown that HCMV infection is associated with increased systolic blood pressure (SBP) in healthy old people [17]. The murine cytomegalovirus (MCMV) has been also associated with an increase in blood pressure. Interestingly, MCMV infection increased the expression of renin in renal cells, suggesting that the renin-mediated increase in the blood volume may contribute to hypertension [18]. The molecular mechanisms underlying the correlation between HCMV infection and elevated blood pressure have not been elucidated. We hypothesized that miRNAs, including virus-encoded miRNA, could be involved in HCMV-mediated hypertension.

MiRNAs are highly conserved, single-stranded, non-coding small RNAs (19‒25 nucleotides in length) that negatively regulate the expression of their target genes at the post-transcriptional level, by directly binding to the 3′-untranslated region (UTR) or 5′-UTR of their target messenger RNAs (mRNAs) [19,20]. Previous studies have shown that several miRNAs are involved in the development of primary hypertension [21,22]. Inhibition of miRNA-126 leads to bleeding, suggesting that miRNA-126 is involved in the maintenance of vascular endothelium integrity [21]. Moreover, miRNA-155 is up-regulated in the plasma of hypertensive subjects, where it inhibits the angiotensin II type I receptor [22]. Notably, Cai et al*.* have shown that the HCMV-encoded miRNA hcmv-miR-UL112 was up-regulated in hypertensive patients, and the blood pressure of the mice significantly increased 9‒12 days after adenoviral transfection of hcmv-miR-UL112 [7]. It has been reported that HCMV can encode at least 26 different mature miRNAs (www.mirbase.org) [23] and can change the miRNA expression profile in the host [19]. Given that hypertension is regulated by a complex miRNA-gene network, it is implausible that hcmv-miR-UL112 is the only miRNA involved in HCMV-mediated regulation of hypertension. Thus, further efforts are needed to identify novel miRNAs involved in HCMV-mediated hypertension.

Our previous studies found that HCMV infection is associated with EH in patients of Kazakh and Han ethnicity in Xinjiang, and is associated with EH progression and target organ damage in patients of Han ethnicity, further supporting the correlation between HCMV and hypertension [24,25]. We thus hypothesized that MCMV may be correlated to hypertension in mice through similar miRNA-induced mechanisms. Therefore, in the present study, we screened differentially expressed miRNAs in mice infected with MCMV, identified the miRNAs potentially involved in hypertension, and experimentally validated our findings to elucidate the role of MCMV in the pathogenesis of hypertension.

## Materials and methods

### Mice

All experiments in the present study were carried out in strict accordance with the recommendations in the Guide for the Care and Use of Laboratory Animals of the National Institutes of Health. The animal model was established as previously reported. A total of 40 C57BL/6 mice (4 weeks of age) were provided by the Institutional Animal Research Committee of Shihezi University School of Medicine. All mice were housed under pathogen-free conditions in individually ventilated cages, placed in a temperature-controlled environment with a 12-h day/12-h night cycle. Mice received food and water *ad libitum*, and were kept at the Shihezi University School of Medicine animal facility. After adaptive feeding for 1 week, the mice were divided into the following two groups: the MCMV infection group (1 × 10^5^ pfu/ml, 1 ml, intraperitoneal injection) and the control group (equivalent volume of saline solution, intraperitoneal injection). All animal experiments were conducted at the Medical College of Shihezi University and performed according to the protocols approved by the Animal Experimental Ethical Inspection of First Affiliated Hospital, Shihezi University School of Medicine (approval number: A2018-038-01).

### Virus and cell culture

MCMV was maintained in NIH 3T3 cells. NIH 3T3 cells were cultured in Dulbecco’s modified Eagle’s medium (DMEM) supplemented with 10% calf serum and 1% antibiotic solution (100 U/ml of penicillin and 100 µg/ml of streptomycin). The virus was collected from the supernatant of infected NIH 3T3 (infection rate: 100%) by centrifugation (15000×***g*** for 20 min) and then further purified with ultracentrifugation (20000 rpm for 1 h) through a 15% sucrose cushion in a virus standard buffer (VSB, 50 mM Tris/HCl pH 7.8, 12 mM KCl, and 5 mM EDTA). The virus pellet was resuspended in 0.5–1.0 ml of VSB, aliquoted, and stored at −80°C. Viral titers were determined with standard plaque assay in NIH 3T3 cells. Both the MCMV Smith strain and NIH 3T3 cells were donated by the Department of Neurovirology, Wuhan Institute of Virology (Hubei, China).

### Blood pressure measurement

The mice were anesthetized using sodium pentobarbital (P3761, Sigma–Aldrich, St. Louis, MO, U.S.A.) through intraperitoneal injection (30 mg/kg), after which they were fixed on the operating table. An incision was made along the midline of the neck and the neck tissues were separated to expose the common carotid artery. The vagus nerves surrounding the carotid artery were separated carefully. A silicone tube (internal diameter: 0.3 mm) was implanted near the artery (diameter: ∼0.5 mm) and a cannula was ligated using 6-0 silk threads to monitor the blood pressure. Then, the carotid artery was put back into position. Finally, the mice were killed by decapitation under pentobarbital anesthesia.

### RNA isolation and sequencing

The total RNA was isolated from thoracic aorta samples using miRNeasy Mini Kit (Qiagen, Germany, 217004). The total RNA isolation from peripheral blood monocytes for sequencing and validation analysis was performed using the miRcute miRNA isolation kit with the enrichment for small RNAs, following the manufacturer’s instructions (TIANGEN, DP501). RNA quality was measured using the Agilent Bioanalyzer 2100 (Agilent Technologies, Santa Clara, CA, U.S.A.). Paired-end libraries were synthesized by using the QIAseq miRNA Library Kit (Qiagen, Germany) following the QIAseq miRNA Library Kit Guide. The products were then purified and enriched by PCR to create the final cDNA library. Purified libraries were quantified by Qubit® 2.0 Fluorometer (Life Technologies, U.S.A.) and validated using the Agilent 2100 bioanalyzer (Agilent Technologies, U.S.A.) to confirm the insert size and calculate the library concentration. A cluster was generated by cBot with the library diluted to 10 pM and then sequenced on the Illumina Hiseq Xten (Illumina, U.S.A.).

The library construction and sequencing were performed by Sinotech Genomics Co., Ltd (Shanghai, China).

### Small RNA-seq data analysis and target prediction

After removing the adoptor sequences and the contaminated reads, the clean reads were processed for letter anlysis. Sequence files (fastq) were mapped to the reference genome (GRCm38) using Bowtie. Small RNA was classified by Unitas software. The miRNA abundance was expressed as counts of exon model per million mapped reads. Differential expression analysis for mRNA was performed using DESeq software. A minimum of two-fold difference between groups and a *P*-value ≤0.05 were used as criteria for the identification of differentially expressed miRNA genes. We then performed gene ontology (GO) enrichment analysis to annotate gene functions, according to three categories: biological process (BP), cellular component (CC), and molecular function (MF). Next, we performed an enrichment analysis using the Kyoto Encyclopedia of Genes and Genomes (KEGG) to systematically analyze, annotate, and visualize gene functions. Potential targets of the differentially expressed miRNAs were predicted using miRanda (http://www.microrna.org/microrna/home.do), miRDB (http://www.mirdb.org/), and TargetScan (http://www.targetscan.org/). Only the genes identified by all three software were selected as target genes.

### Quantitative reverse transcription polymerase chain reaction

The integrity and purity of total RNA samples were evaluated using PCR and agarose gel electrophoresis. Total RNA samples of suitable quality were reverse transcribed into cDNA using the miRcute Plus miRNA First-Strand cDNA kit (TIANGEN, KR211). The qPCR conditions were the following (Supplementary Table S1): 95°C for 15 min; 5 cycles at 94°C for 20 s, 65°C for 30 s, and 72°C for 34 s; 45 cycles at 94°C for 20 s and 60°C for 34 s. All primers were synthesized by Shanghai Living Creature (China) (Supplementary Table S2). Gene expression levels were calculated using the 2^−ΔΔ*C*_t_^ method [26].

### Plasmid construction and dual-luciferase activity assay

The full-length 3′-UTR of the endothelin A receptor (*Ednra*) gene was amplified by PCR and cloned downstream the pmirGLO Luciferase (Shanghai GenePharma Co., Ltd, China) multiple cloning site by digestion with the SpeI and HindIII restriction enzymes. The resulting construct was called pEdnra-wild-type (Wt). The miR-1929-3p binding site on the Ednra mRNA was mutated and cloned in the pmirGLO Luciferase plasmid to create the pEdnra-Mutant (Mut) vector. The Wt and Mut vectors were each transfected with and without miR-1929-3p into the HEK293 cells. Additionally, a dual-luciferase reporter gene assay was performed using the dual-luciferase reporter assay system (Shanghai GenePharma Co., Ltd, China).

The mmu-miR-1929-3p mimics, corresponding negative control and siRNA targeting Ednra were synthesized from GenePharma (Shanghai GenePharma Co., Ltd, China). Ednra sense 5′-CCTGGCAGGAAACAATGTCAAAGTGGCCAAATGAGCTGATTGTGTTAAGTGAGATGTAGTTACACC-3′ antisense 5′-TCGAGGTGTAACTACATCTCACTTAACACAATCAGCTCATTTGGCCACTTTGACATTGTTTCCTGCCAGGAGCT-3′; si-Ednra sense 5′-CGCAGAACAUCAAAGAAGATT-3′, antisense 5′-UCUUCUUUGAUGUUCUGCGTT-3′; negative control siRNA sense 5′-UUCUCCGAACGUGUCACGUTT-3′, antisense 5′-ACGUGACACGUUCGGAGAATT-3′.

### Statistical analysis

The SPSS 21.0 statistical software (IBM Corp., Armonk, NY, U.S.A.) was used for statistical analysis. Data were presented throughout the study as mean ± standard deviation. The *t* test was used for comparing the differences between the two groups. A *P*-value <0.05 indicated a statistically significant difference.

## Results

### MCMV infection raises blood pressure in C57BL/6 mice

**Figure 1:**
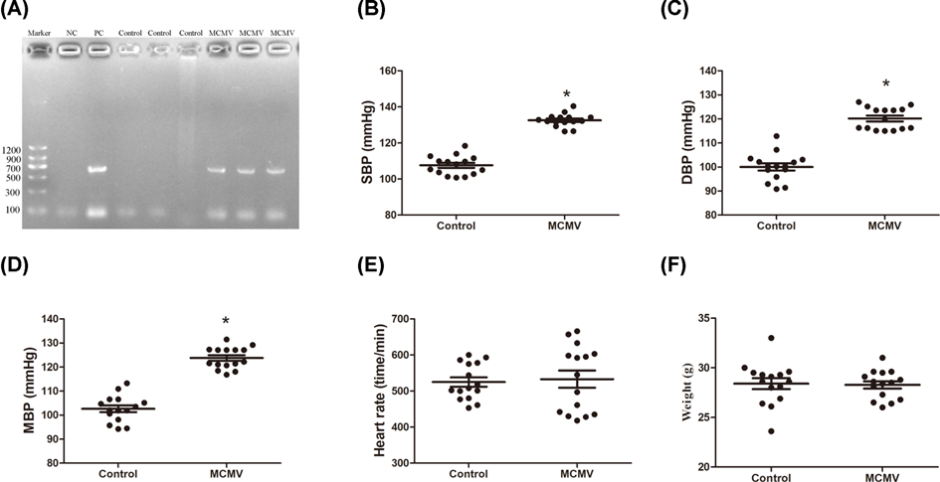
The MCMV infection raised blood pressure in C57BL/6 mice (**A**) PCR analysis of MCMV IE gene in the thoracic aorta of male C57BL/6 mice. NC, negative control group. PC, positive control group. M, marker. 1∼4, thoracic aorta of different mice infected with MCMV for 21 days. (**B**) SBP. (**C**) Diastolic blood pressure. (**D**) Mean arterial pressure. (**E**) Heart rate. (**F**) Weight. The data are expressed as the means ± SEM, (*n*=15), *P<0.05 vs. the control group.

To confirm the MCMV infection, we measured with quantitative reverse transcription polymerase chain reaction (qRT-PCR) the MCMV IE1 gene expression in the thoracic aorta of mice infected with the virus (MCMV IE Forward primer: 5′-ATC AAT CAG CCA TCA ACT CTG CTA CCA CAC-3′; MCMV IE Reverse primer: 5′-ATG GTG AAG CTA TCA AAG ATG TGC ATC TCA-3′). The results confirmed that the infection was successful (Figure 1A). Then, we measured the blood pressure and observed that it was significantly higher in mice infected with MCMV after 10 weeks (*P*<0.01; Figure 1B–D). However, the heart rate and weight were not significantly different between the two groups (Figure 1E,F).

### Differential miRNA expression upon MCMV infection

**Figure 2:**
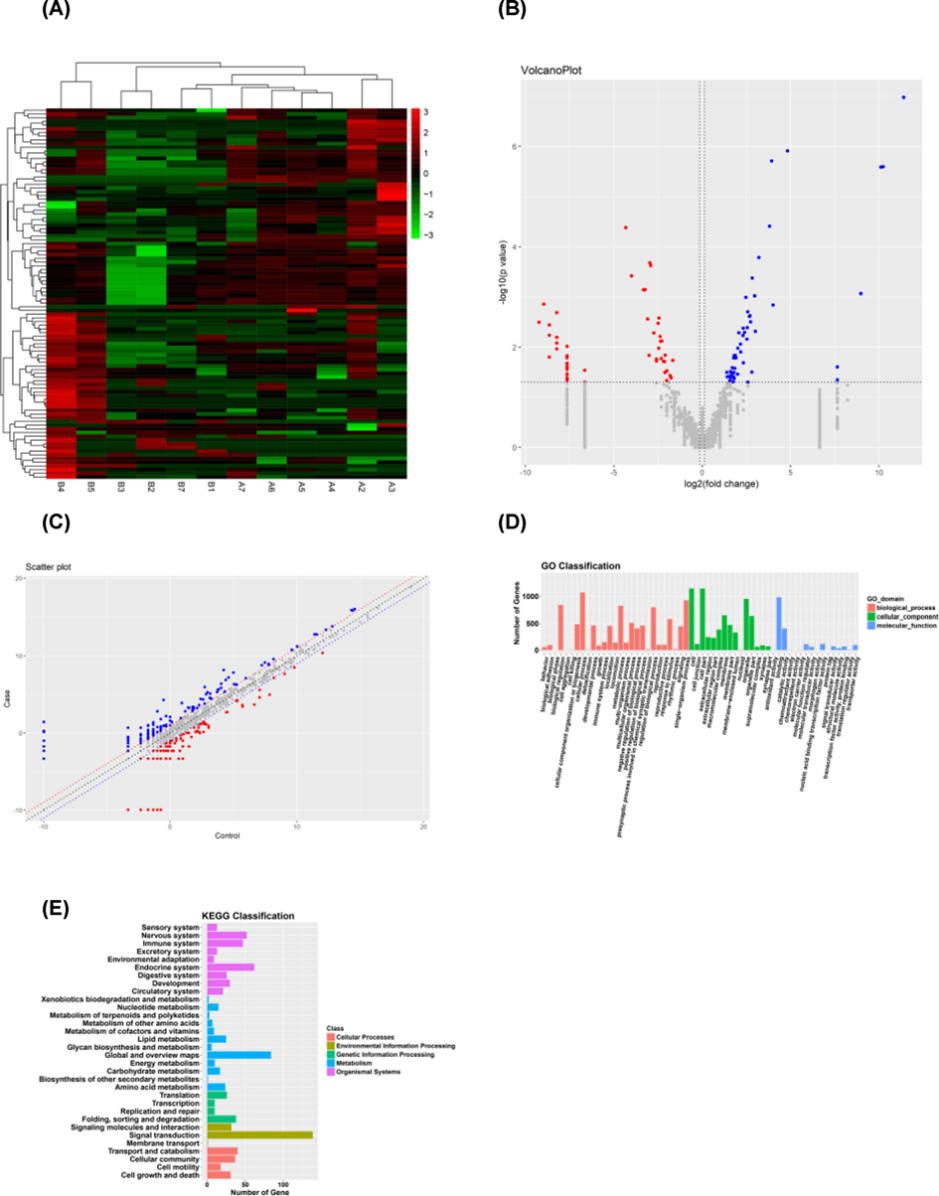
MiRNA expression changes by MCMV infection and bioinformatics analysis x-axis: Sample.ID. the gray lines represent a log2 difference of 1 in the 2C. (**A**) Heat map of all 118 significantly changed miRNAs in the peripheral blood identified by sequencing (*n*=6). A2∼A7: MCMV group; B1∼B7: Control group. Red (up) and green (down) indicate relative fold-changes for each comparison. (**B**) Scatter plot of mean expression between MCMV group and control group. (**C**) Volcano map of differentially expressed miRNAs. (**D**) Top 20 genes for GO function enrichment analysis of target genes regulated by differential miRNAs. (**E**) KEGG Pathway enrichment analysis of hypertension-related target genes (top 20).

We compared miRNA expression profiles in the peripheral blood of mice infected with MCMV with uninfected mice using a high-throughput sequencing approach. We found 118 significantly differentially expressed miRNAs, of which 91 were up-regulated and 27 were down-regulated (absolute value fold-change ≥ 1, *P*<0.05; Figure 2A and Supplementary Table S3). Scatter plots and volcano plots of the standardized expression data showed the miRNA expression trend between the two groups (Figure 2B,C). The GO function enrichment analysis and the KEGG enrichment analysis of the top 20 target genes regulated by differentially expressed miRNAs are shown in Figure 2D (Supplementary Table S8) and 2E (Supplementary Table S9), respectively.

### Screening of hypertension-related miRNAs and target genes prediction

**Figure 3:**
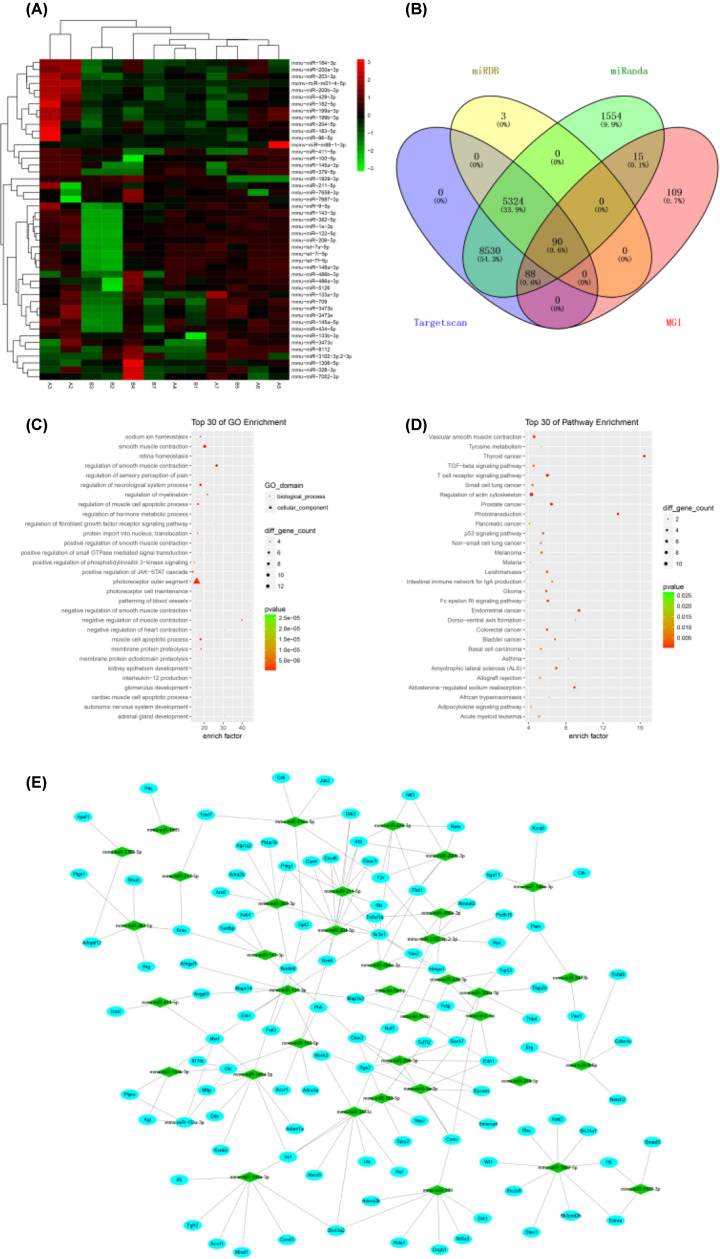
Screening of hypertension-related miRNAs and prediction of target genes x-axis: Sample.ID. (**A**) Heat map of all 47 significantly changed miRNAs in the peripheral blood identified by sequencing (*n*=6). A2∼A7: MCMV group; B1∼B7: Control group. Red (up) and green (down) indicate relative fold-changes for each comparison. (**B**) Venn diagrams of target genes and mouse hypertension-related genes predicted by three databases of differentially expressed miRNAs. (**C**) Top 30 genes for GO function enrichment analysis of hypertension-related target genes regulated by differentially expressed miRNAs. (**D**) KEGG Pathway enrichment analysis of hypertension-related target genes (top 30). (**E**) MCMV-induced hypertension-related differential expression of miRNAs and target gene network regulation.

We further analyzed the small RNA-seq data to identify differentially expressed miRNAs involved in hypertension. After removing the unknown and weak signal samples from each group, we performed cluster analysis and found a cluster containing 36 up-regulated and 11 down-regulated miRNAs (Figure 3A and Supplementary Table S4). Then, we predicted the miRNA target genes using bioinformatics methods. We found 90 hypertension-related target genes that were potentially targeted by 32 differentially regulated miRNAs, among which 26 were up-regulated and 6 down-regulated (Figure 3B) (Supplementary Table S5). The GO function and the KEGG pathway enrichment analysis of the top 30 hypertension-related target genes are shown in Figure 3C,D and Supplementary Table S6).

To further identify miRNAs closely related to hypertension, we performed a ranking analysis between genes correlated with hypertension and high blood pressure with 29 miRNAs, 2 of them of CMV origin (Table 1). Then, we built an miRNA–mRNA regulatory interaction network (Figure 3E and Supplementary Table S7) and selected 9 candidate miRNAs for experimental validation of their role in MCMV-induced hypertension (Table 2).

**Table 1:** Interaction analysis of MCMV-induced hypertension-related differential expression mRNA target genes and GCBI (top 20 diseases)

Up-regulated miRNAs	Down-regulated miRNAs
mmu-miR-211-5p	mmu-miR-328-3p
mmu-miR-379-5p	mmu-miR-486a-3p
mmu-miR-204-5p	mmu-miR-1929-3p
mmu-miR-183-5p	
mmu-miR-143-3p	
mmu-miR-133b-3p	
mmu-miR-182-5p	
mmu-miR-411-5p	
mmu-miR-1a-3p	
mmu-miR-203-3p	
mmu-miR-429-3p	
mmu-miR-3473c	
mmu-miR-145a-5p	
mmu-miR-145a-3p	
mmu-miR-200b-3p	
mmu-miR-709	
mmu-miR-206-3p	
mmu-miR-9-5p	
mmu-miR-148a-3p	
mmu-miR-200a-3p	
mmu-miR-133a-3p	
mmu-let-7f-5p	
mmu-let-7i-5p	
mmu-let-7a-5p	
mcmv-miR-m01-4-5p	
mcmv-miR-m88-1-3p	

Abbreviation: GCBI, Gene-cloud of Biotechnology Information.

**Table 2:** Interaction analysis of MCMV-induced hypertension-related mRNA target genes of differentially expressed miRNAs and GCBI

Up-regulated miRNAs	Target genes associated with hypertension	Down-regulated miRNAs	Target genes associated with hypertension
mmu-miR-133b-3p	*Agt*	mmu-miR-328-3p	*Nr3c1, Tcf7l2*
mmu-miR-143-3p	*Asb4*	mmu-miR-1929-3p	*Ednra, Smad9*
mmu-miR-182-5p	*Adra2a, Rgs2*		
mmu-miR-204-5p	*Nr3c1, Prkg1, Nedd4l*		
mmu-miR-211-5p	*Nr3c1, Prkg1*		
mcmv-miR-m01-4-5p	*Slc12a6*		
mcmv-miR-m88-1-3p	*KLHL3*		

Abbreviations: Adra2a, adrenergic receptor, α 2a; Agt, angiotensinogen (serpin peptidase inhibitor, clade A, member 8); Asb4, ankyrin repeat and SOCS box-containing 4; *Ednra*, endothelin receptor type A; GCBI, Gene-cloud of Biotechnology Information; KLHL3, Kelch-like 3; Nedd4l, neural precursor cell expressed, developmentally down-regulated gene 4-like; Nr3c1, nuclear receptor subfamily 3, group C, member 1; Prkg1, protein kinase, cGMP-dependent, type I; Rgs2, regulator of G-protein signaling 2; Slc12a6, solute carrier family 12 (potassium/chloride transporters), member 6; Smad9, SMAD family member 9; Tcf7l2, transcription factor 7 like 2, T-cell specific, HMG box.

### Validation of miRNA differential expression with qRT-PCR

**Figure 4:**
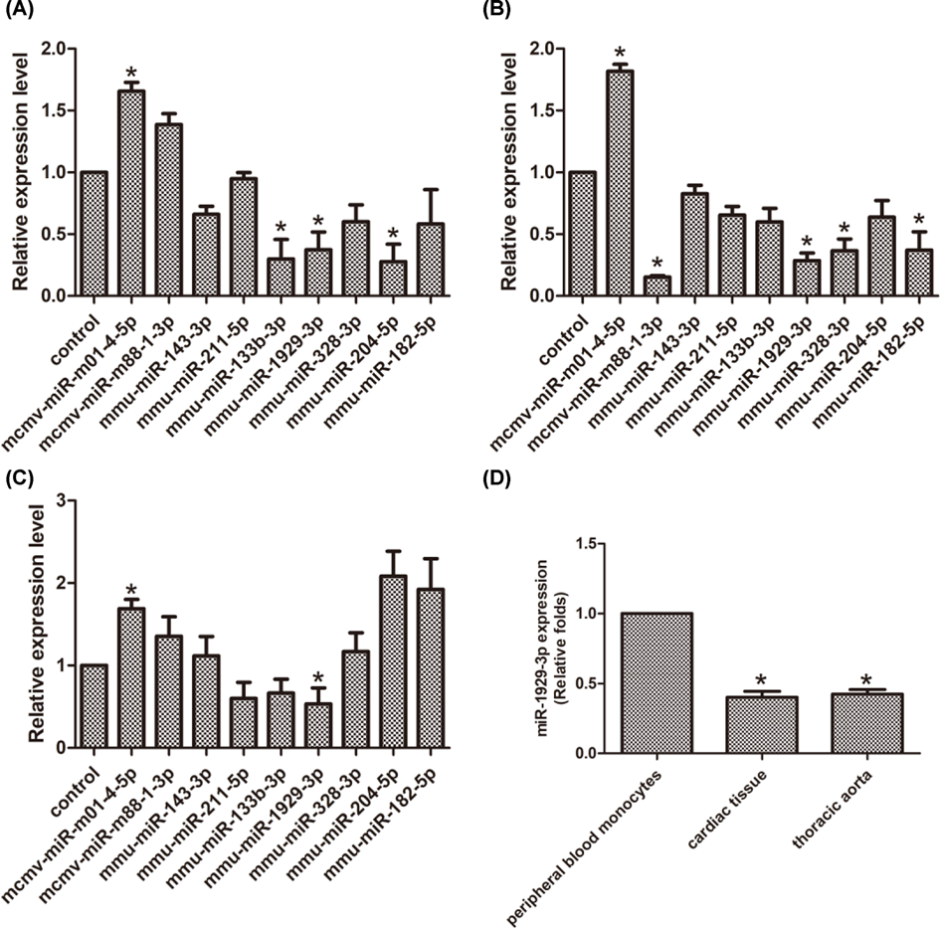
The value of each group of differentially expressed miRNA in MCMV-infected C57BL/6 mice Control: value observed with uninfected control. (**A**–**C**) Differential expression of miRNA in peripheral blood monocytes, cardiac tissue and the thoracic aorta by RT-qPCR. The value at each group was normalized to the value observed with control, which was set to 1. (**D**) mmu-miR-1929-3p expression in peripheral blood monocytes, cardiac tissue and the thoracic aorta by RT-qPCR. The value at each group was normalized to the value observed with peripheral blood monocytes, which was set to 1. The data are expressed as the means ± SEM, (*n*=15), *P<0.05 vs. the control group or peripheral blood monocytes group.

qRT-PCR was used to validate the expression levels of the nine differentially expressed miRNAs in independent samples from different tissues (*n*=10). We isolated monocytes by performing Ficoll density gradient centrifugation and found that in peripheral blood monocytes of mice infected with MCMV [27], mcmv-miR-m01-4-5p increased, while mmu-miR-133b-3p, mmu-miR-1929-3p, and mmu-miR-204-5p decreased (Figure 4A). In the thoracic aorta, mcmv-miR-m01-4-5p increased and mmu-miR-1929-3p decreased (Figure 4B). In the cardiac tissue mcmv-miR-m01-4-5p increased, while mcmv-miR-m88-1-3p, mmu-miR-1929-3p, mmu-miR-328-3p, and mmu-miR-182-5p decreased (Figure 4C). Thus, mmu-miR-1929-3p was the only host-derived miRNA consistently down-regulated in all three tissues. Moreover, we observed that the expression levels of mmu-miR-1929-3p in the peripheral blood monocytes were significantly higher than those in the thoracic aorta and cardiac tissue (Figure 4D). At the same time, in our experimental validation using MCMV to infect NIH3T3 cells *in vitro*, we still found that MCMV infection is able to down-regulate the expression of mmu-miR-1929-3p in infected cells (Supplementary Figure S1B).

### *Ednra* is a target of mmu-miR-1929-3p

**Figure 5:**
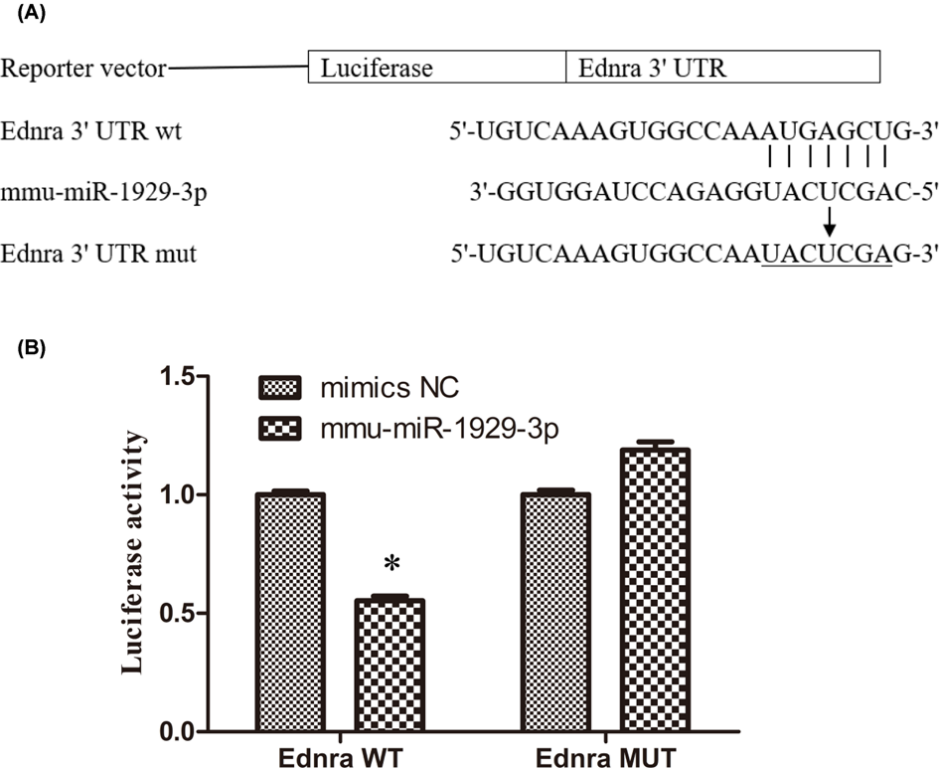
*Ednra* is determined to be a target of mmu-miR-1929-3p NC, negative control. (**A**) Sequences of the 3′-UTR of the *Ednra* mRNA binding with mmu-miR-1929-3p. *Ednra*, endothelin receptor type A. (**B**) Luciferase activity in the Ednra-WT and Ednra-MUT group. *P<0.05 vs. the mimics NC group.

Using a bioinformatics approach, we found that endothelin receptor type A (*Ednra*) could be a potential target of mmu-miR-1929-3p (Figure 5A). Dual-luciferase reporter assay showed that the luciferase activity significantly dropped when we transfected the mednra-miR1929-WT vector (*P*<0.05), while no significant difference was observed upon transfecting the mednra-miR1929-MUT vector or an NC mimic (*P*>0.05) (Figure 5B). At the same time, we found that in vascular tissues, the expression of *Ednra* mRNA in the MCMV infection group was higher than that in the control group (Supplementary Figure S1A). Thus, we experimentally validated that *Ednra* is a target of mmu-miR-1929-3p.

## Discussion

In the present study, we observed that MCMV infection caused the differential expression of various miRNA, originating both from the host and the virus, which could play a role in the pathogenesis of hypertension. In particular, we found that the MCMV-encoded mcmv-miR-m01-4-5p miRNA increased, while the host-encoded mmu-miR-1929-3p miRNA decreased. However, as the target genes regulated by mcmv-miR-m01-4-5p did not rank among the top ten hypertension-related genes in the bioinformatic analysis, we focused on mmu-miR-1929-3p for the subsequent analysis. CMV infection is usually latent, although it undergoes periodic reactivation events, by bypassing the host immune response [28]. Although HCMV infection is usually asymptomatic, congenital HCMV infection can cause severe birth defects, including hearing loss, cognitive impairment, and microcephaly [16,29]. As MCMV is a natural mouse pathogen, it is not necessary to manipulate the host. Thus, the study of MCMV provides a unique model for studying *in vivo* the biological mechanism of viruses of the CMV genus. MCMV has been extensively used in cardiovascular studies [30]. Much of the information derived from studies using this model has been translated into clinical practice [31]. A previous study has shown that CMV infection causes an increase in arterial blood pressure [18]. Similarly, we reported that CMV infection may be correlated to hypertension in patients belonging to two different ethnicities living in the Xinjiang region [32]. In this study, we infected C57BL/6 mice with MCMV to establish a model presenting elevated blood pressure. Our results confirmed that, after 10 weeks of infection, the blood pressure is significantly higher in infected mice than that in the uninfected mice.

It has been widely recognized that miRNAs play an important role in regulating cardiovascular functions [33,34]. Patients with coronary heart disease are characterized by a high serum level of miR-486, miR-92a, and miR-122 [35]. Moreover, the level of circulating miR-126 is increased in patients with acute myocardial infarction and angina [36], while plasma miR-126 levels are down-regulated in patients with diabetes [37], heart failure [38], and cancer [39]. Recently, miR-126 has been identified as an effective biomarker for endothelial cell detection and purification [40]. Many studies reported a functional role for miRNAs in hypertension. According to *in vitro* studies, CMV-encoded miRNAs play an important regulatory role in viral replication, but the association between host-encoded miRNAs, HCMV-encoded miRNAs, and hypertension remain unclear. Here, we identified 118 differentially expressed miRNAs. Among them, the expression of two miRNA encoded by MCMV was up-regulated. These results suggest that both MCMV- and host-encoded miRNA may be involved in the insurgence of MCMV-mediated hypertension.

MiRNAs are known to be effective regulators of gene expression, by binding to target mRNA, blocking their translation by degrading the mRNA or inhibiting the translation process [34]. ACE inhibitors and angiotensin 1 receptor blockers have been reported to partially reverse vascular dysfunction and normalize gene expression in miR-143/145-deleted mice [41]. Marques et al*.* demonstrated that miR-181a-5p binds to the 3′-UTR of the renin mRNA to reduce its gene expression. In hypertensive individuals, high expression of renin mRNA is accompanied by a decreased expression of miR-181a in the kidney [42]. In rats with hypertension complicated with heart failure, miR-16, miR-20b, miR-93, miR-106b, miR-223, and miR-423-5p circulating levels increases significantly [43]. By inhibiting miR-208a, the circulating levels of these miRNA decrease, suggesting that plasma miRNA changes can be used as biomarkers of disease progression and manipulated to treat hypertension in the context of heart disease [43]. In this study, we validated with qRT-PCR the expression of mmu-miR-1929-3p and mcmv-miR-m01-4-5p in peripheral blood monocytes, blood vessels, and myocardial tissues samples. Although the exact role of viral-encoded miRNAs is not known in many cases, it has been proposed that they could be a common mechanism by which viruses modulate transcriptional changes in the host and their genome during toxic infection and/or incubation. For instance, the simian virus 40-encoded miR-s1 miRNA mediates the *cis*-degradation of cytotoxic t-antigen mRNA, which encodes for a major viral regulator, in a process thought to help to evade the host immune response [44]. The HCMV-encoded miR-ul112-3p targets the 3′-UTR of gene 72 (IE72), an early major *trans*-activator gene. The removal of miR-ul112-3p target sites in the IE72 mRNA increased IE72 expression [45]. One of the differentially expressed miRNAs detected in this study was mcmv-miR-m01-4, which is derived from a pre-miRNA located in the 3′-UTR of the m01 transcript and accumulates to detectable levels *in vivo* [46]. However, we did not find any potential hypertension-related target genes. In contrast, for mmu-miR-1929-3p, we predicted a hypertension-related target gene, *Ednra*, and experimentally validated the finding with the dual-luciferase assay.

In mammals, the endothelin signaling system (ligand plus receptor) consists of three ligands (encoded by *EDN1, EDN2*, and *EDN3*) and two receptors (encoded by *Ednra* and *Ednrb*), belonging to the seven-fold transmembrane domain receptor family of G protein-conjugated receptors [46]. The *Ednra* gene spans over 40 kb and contains 8 exons and 7 introns [47]. In hypertensive patients, *Ednra* increases vasoconstriction tension and may be associated with an increase in endothelin-1 (ET-1) [48]. ET-1 is the most effective vasoconstrictor in the human cardiovascular system and has very long-lasting effects. In the present study, we showed that mmu-miR-1929-3p targets *Ednra* mRNA and thus might be involved in MCMV-induced hypertension. Future studies are required to identify potential biomarkers of CMV-mediated hypertension.

A previous study identified different virus- and host-encoded miRNAs whose target genes are related to the inflammatory response, vasoconstriction, and vascular remodeling (although their identified target genes are not among the top ten genes involved in the incidence of hypertension) [34]. The discrepancy with our results can be explained by various factors, such as the use of different models, research objects, research methods, and CMV specificity in different species. However, both the present study and our support after the idea that MCMV infection promotes the development of hypertension through the miRNA-mediated regulation. Both studies also suggest that antiviral therapy can be a novel approach for the prevention and treatment of hypertension.

The present study presents some limitations. In particular, the effect of mmu-miR-1929-3p in the occurrence and development of hypertension and target organ damage still need to be further validated *in vivo* and elucidated at the cellular and molecular level.

In conclusion, we identified mmu-miR-1929-3p as a novel miRNA down-regulated in mice infected with MCMV. We also experimentally validated that *Ednra* may be the target gene of mmu-miR-1929-3p. Thus, mmu-miR-1929-3p can be promising drug targets and/or potential biomarkers for the prevention and management of hypertension.

## Supplementary Material

Supplementary Figure S1 and Tables S1-S9

## Data Availability

The data associated with the present paper are stored in NCBI SRA (accession number: PRJNA679174). Other data that were used to support the findings of the present study are included within the supplementary information files.

## Abbreviations

ACE: angiotensin
CMV: cytomegalovirus
*Ednra*: endothelin A receptor
EH: essential hypertension
ET-1: endothelin-1
GO: gene ontology
HCMV: human cytomegalovirus
KEGG: Kyoto Encyclopedia of Genes and Genomes
MCMV: murine cytomegalovirus
miRNA: microRNA
mRNA: messenger RNA
PFU: plaque forming unit
qRT-PCR: quantitative reverse transcription polymerase chain reaction
UTR: untranslated region
VSB: virus standard buffer

## References

[1] Oscullo G. et al. (2019) Resistant/refractory hypertension and sleep apnoea: current knowledge and future challenges. J. Clin. Med. 8(11), 10.3390/jcm8111872 PMC6912579 31694223

[2] Romaine S.P., Charchar F.J., Samani N.J. and Tomaszewski M. (2016) Circulating microRNAs and hypertension–from new insights into blood pressure regulation to biomarkers of cardiovascular risk. Curr. Opin. Pharmacol. 27, 1–7 10.1016/j.coph.2015.12.002 26827149

[3] Hamet P., Pausova Z., Adarichev V., Adaricheva K. and Tremblay J. (1998) Hypertension: genes and environment. J. Hypertens. 16, 397–418 10.1097/00004872-199816040-00001 9797185

[4] Carretero O.A. and Oparil S. (2000) Essential hypertension. Part I: definition and etiology. Circulation 101, 329–335 10.1161/01.CIR.101.3.329 10645931

[5] Kunes J. and Zicha J. (2009) The interaction of genetic and environmental factors in the etiology of hypertension. Physiol. Res. 58, S33–S41 10.33549/physiolres.931913 20131935

[6] Nemecz M., Alexandru N., Tanko G. and Georgescu A. (2016) Role of MicroRNA in endothelial dysfunction and hypertension. Curr. Hypertens. Rep. 18, 87 10.1007/s11906-016-0696-8 27837398 PMC7102349

[7] Li S. et al. (2011) Signature microRNA expression profile of essential hypertension and its novel link to human cytomegalovirus infection. Circulation 124, 175–184 10.1161/CIRCULATIONAHA.110.012237 21690488

[8] Cannon M.J., Schmid D.S. and Hyde T.B. (2010) Review of cytomegalovirus seroprevalence and demographic characteristics associated with infection. Rev. Med. Virol. 20, 202–213 10.1002/rmv.655 20564615

[9] Haarala A. et al. (2012) Relation of high cytomegalovirus antibody titres to blood pressure and brachial artery flow-mediated dilation in young men: the Cardiovascular Risk in Young Finns Study. Clin. Exp. Immunol. 167, 309–316 10.1111/j.1365-2249.2011.04513.x 22236008 PMC3278698

[10] Li C., Samaranayake N.R., Ong K.L., Wong H.K. and Cheung B.M. (2012) Is human cytomegalovirus infection associated with hypertension? The United States National Health and Nutrition Examination Survey 1999-2002. PLoS ONE 7, e39760 10.1371/journal.pone.0039760 22768311 PMC3388091

[11] Du Y., Zhang G. and Liu Z. (2018) Human cytomegalovirus infection and coronary heart disease: a systematic review. Virol. J. 15, 31 10.1186/s12985-018-0937-3 29409508 PMC5801777

[12] Murphy E. et al. (2003) Coding potential of laboratory and clinical strains of human cytomegalovirus. Proc. Natl. Acad. Sci. U.S.A. 100, 14976–14981 10.1073/pnas.2136652100 14657367 PMC299866

[13] Gatherer D. et al. (2011) High-resolution human cytomegalovirus transcriptome. Proc. Natl. Acad. Sci. U.S.A. 108, 19755–19760 10.1073/pnas.1115861108 22109557 PMC3241806

[14] Nogalski M.T., Collins-McMillen D. and Yurochko A.D. (2014) Overview of human cytomegalovirus pathogenesis. Methods Mol. Biol. 1119, 15–28 10.1007/978-1-62703-788-4_2 24639215

[15] Pass R.F., Fowler K.B., Boppana S.B., Britt W.J. and Stagno S. (2006) Congenital cytomegalovirus infection following first trimester maternal infection: symptoms at birth and outcome. J. Clin. Virol. 35, 216–220 10.1016/j.jcv.2005.09.015 16368262

[16] Swanson E.C. and Schleiss M.R. (2013) Congenital cytomegalovirus infection: new prospects for prevention and therapy. Pediatr. Clin. North Am. 60, 335–349 10.1016/j.pcl.2012.12.008 23481104 PMC3807860

[17] Firth C. et al. (2016) Cytomegalovirus infection is associated with an increase in systolic blood pressure in older individuals. QJM 109, 595–600 10.1093/qjmed/hcw026 27071749 PMC5027953

[18] Cheng J. et al. (2009) Cytomegalovirus infection causes an increase of arterial blood pressure. PLoS Pathog. 5, e1000427 10.1371/journal.ppat.1000427 19436702 PMC2673691

[19] Sorel O. and Dewals B.G. (2016) MicroRNAs in large herpesvirus DNA genomes: recent advances. Biomol. Concepts 7, 229–239 10.1515/bmc-2016-0017 27544723

[20] Grey F. et al. (2010) A viral microRNA down-regulates multiple cell cycle genes through mRNA 5¢UTRs. PLoS Pathog. 6, e1000967 10.1371/journal.ppat.1000967 20585629 PMC2891821

[21] Levy E., Spahis S., Bigras J.L., Delvin E. and Borys J.M. (2017) The epigenetic machinery in vascular dysfunction and hypertension. Curr. Hypertens. Rep. 19, 52 10.1007/s11906-017-0745-y 28540644

[22] Ceolotto G. et al. (2011) Interplay between miR-155, AT1R A1166C polymorphism, and AT1R expression in young untreated hypertensives. Am. J. Hypertens. 24, 241–246 10.1038/ajh.2010.211 20966899

[23] Meshesha M.K. et al. (2012) The microRNA transcriptome of Human Cytomegalovirus (HCMV). Open Virol. J. 6, 38–48 10.2174/1874357901206010038 22715351 PMC3377890

[24] Li Z. et al. (2017) High anti-human cytomegalovirus antibody levels are associated with the progression of essential hypertension and target organ damage in Han Chinese population. PLoS ONE 12, e0181440 10.1371/journal.pone.0181440 28837559 PMC5570371

[25] Feng Q. et al. (2018) Unexpected role of the human cytomegalovirus contribute to essential hypertension in the Kazakh Chinese population of Xinjiang. Biosci. Rep. 38(3), 10.1042/BSR20171522 PMC6019381 29752343

[26] Livak K.J. and Schmittgen T.D. (2001) Analysis of relative gene expression data using real-time quantitative PCR and the 2(-Delta Delta C(T)) Method. Methods 25, 402–408 10.1006/meth.2001.1262 11846609

[27] Zhou L. et al. (2012) Impact of human granulocyte and monocyte isolation procedures on functional studies. Clin. Vaccine Immunol. 19, 1065–1074 10.1128/CVI.05715-11 22552601 PMC3393372

[28] Sweet C. (1999) The pathogenicity of cytomegalovirus. FEMS Microbiol. Rev. 23, 457–482 10.1111/j.1574-6976.1999.tb00408.x 10422262

[29] Reddehase M.J., Podlech J. and Grzimek N.K. (2002) Mouse models of cytomegalovirus latency: overview. J. Clin. Virol. 25, S23–S36 10.1016/S1386-6532(02)00087-2 12361754

[30] Tang-Feldman Y.J. et al. (2013) Murine cytomegalovirus (MCMV) infection upregulates P38 MAP kinase in aortas of Apo E KO mice: a molecular mechanism for MCMV-induced acceleration of atherosclerosis. J. Cardiovasc. Transl. Res. 6, 54–64 10.1007/s12265-012-9428-x 23192592 PMC4591060

[31] Crough T. and Khanna R. (2009) Immunobiology of human cytomegalovirus: from bench to bedside. Clin. Microbiol. Rev. 22, 76–98, Table of contents 10.1128/CMR.00034-08 19136435 PMC2620639

[32] Tang N. et al. (2014) Human cytomegalovirus infection is associated with essential hypertension in Kazakh and Han Chinese populations. Med. Sci. Monit. 20, 2508–2519 25448630 10.12659/MSM.892861 PMC4262054

[33] Viereck J. and Thum T. (2017) Circulating noncoding RNAs as biomarkers of cardiovascular disease and injury. Circ. Res. 120, 381–399 10.1161/CIRCRESAHA.116.308434 28104771

[34] Small E.M. and Olson E.N. (2011) Pervasive roles of microRNAs in cardiovascular biology. Nature 469, 336–342 10.1038/nature09783 21248840 PMC3073349

[35] Niculescu L.S. et al. (2015) MiR-486 and miR-92a identified in circulating HDL discriminate between stable and vulnerable coronary artery disease patients. PLoS ONE 10, e0140958 10.1371/journal.pone.0140958 26485305 PMC4617647

[36] Xue S. et al. (2019) Circulating miR-17-5p, miR-126-5p and miR-145-3p are novel biomarkers for diagnosis of acute myocardial infarction. Front. Physiol. 10, 123 10.3389/fphys.2019.00123 30833907 PMC6387945

[37] Liu Y. et al. (2014) The role of circulating microRNA-126 (miR-126): a novel biomarker for screening prediabetes and newly diagnosed type 2 diabetes mellitus. Int. J. Mol. Sci. 15, 10567–10577 10.3390/ijms150610567 24927146 PMC4100169

[38] Schneider S. et al. (2018) Plasma levels of microRNA-21, -126 and -423-5p alter during clinical improvement and are associated with the prognosis of acute heart failure. Mol. Med. Rep. 17, 4736–4746 29344661 10.3892/mmr.2018.8428

[39] Huang W., Lin J. and Zhang H. (2016) miR-126: a novel regulator in colon cancer. Biomed. Rep. 4, 131–134 10.3892/br.2015.549 26893826 PMC4734020

[40] Miki K. et al. (2015) Efficient detection and purification of cell populations using synthetic microRNA switches. Cell Stem Cell 16, 699–711 10.1016/j.stem.2015.04.005 26004781

[41] Boettger T. et al. (2009) Acquisition of the contractile phenotype by murine arterial smooth muscle cells depends on the Mir143/145 gene cluster. J. Clin. Invest. 119, 2634–2647 10.1172/JCI38864 19690389 PMC2735940

[42] Marques F.Z. et al. (2011) Gene expression profiling reveals renin mRNA overexpression in human hypertensive kidneys and a role for microRNAs. Hypertension 58, 1093–1098 10.1161/HYPERTENSIONAHA.111.180729 22042811

[43] Dickinson B.A. et al. (2013) Plasma microRNAs serve as biomarkers of therapeutic efficacy and disease progression in hypertension-induced heart failure. Eur. J. Heart Fail. 15, 650–659 10.1093/eurjhf/hft018 23388090

[44] Sullivan C.S., Grundhoff A.T., Tevethia S., Pipas J.M. and Ganem D. (2005) SV40-encoded microRNAs regulate viral gene expression and reduce susceptibility to cytotoxic T cells. Nature 435, 682–686 10.1038/nature03576 15931223

[45] Grey F., Meyers H., White E.A., Spector D.H. and Nelson J. (2007) A human cytomegalovirus-encoded microRNA regulates expression of multiple viral genes involved in replication. PLoS Pathog. 3, e163 10.1371/journal.ppat.0030163 17983268 PMC2048532

[46] Dölken L. et al. (2007) Mouse cytomegalovirus microRNAs dominate the cellular small RNA profile during lytic infection and show features of posttranscriptional regulation. J. Virol. 81, 13771–13782 10.1128/JVI.01313-07 17942535 PMC2168864

[47] Hosoda K. et al. (1992) Organization, structure, chromosomal assignment, and expression of the gene encoding the human endothelin-A receptor. J. Biol. Chem. 267, 18797–18804 1326535

[48] Campia U., Cardillo C. and Panza J.A. (2004) Ethnic differences in the vasoconstrictor activity of endogenous endothelin-1 in hypertensive patients. Circulation 109, 3191–3195 10.1161/01.CIR.0000130590.24107.D3 15148269

