# Supplementary material for: Screening and validation of differentially expressed microRNAs and target genes in hypertensive mice induced by cytomegalovirus infection
Source: Biosci Rep. 2020 Dec 10;40(12):BSR20202387. doi: 10.1042/BSR20202387 (PMC7729292; doi:10.1042/BSR20202387)
Supplement: Supplementary Figure S1 and Tables S1-S9 [file BSR-2020-2387_supp.pdf]

Supplementary Figure 1. The expression levels of *Ednra* in tissues and miR-1929-3p in cells

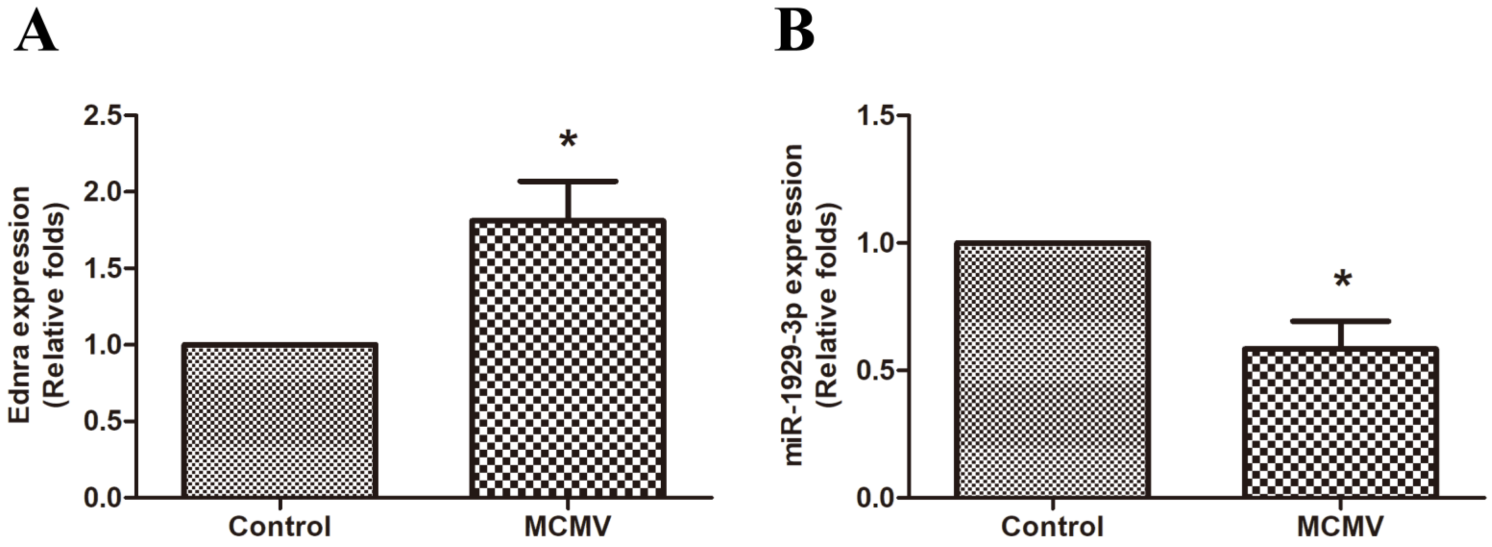

Supplementary Table 1. The qRT-PCR system

Reaction Component	Concentration	Volume(μL)
2×miRcute Plus miRNA PreMix（SYBR＆ROX）	2×	10
Forward primer（U6）	200 nM	0.4
Reverse primer	200 nM	0.4
ddH2O	-	8.2
miRNA the first chain cDNA	-	1
Total Volume	-	20

Supplementary Table 2. The primer sequences for qRT-PCR

microRNA	Sequence	Accession number
mmu-miR-204-5p	5'-UUCCCUUUGUCAUCCUAUGCCU-3'	MIMAT0000237
mmu-miR-182-5p	5'-UUUGGCAAUGGUAGAACUCACACCG-3'	MIMAT0000211
mmu-miR-143-3p	5'-UGAGAUGAAGCACUGUAGCUC-3'	MIMAT0000247
mmu-miR-211-5p	5'-UUCCCUUUGUCAUCCUUUGCCU-3'	MIMAT0000668
mmu-miR-133b-3p	5'-UUUGGUCCCCUUCAACCAGCUA-3'	MIMAT0000769
mmu-miR-328-3p	5'-CUGGCCCUCUCUGCCCUUCCGU-3'	MIMAT0000565
mmu-miR-1929-3p	5'-CAGCUCAUGGAGACCUAGGUGG-3'	MIMAT0022729
mcmv-miR-m01-4-5p	5'-UCCUAUGCUAACACGUGCGCGUG-3'	MIMAT0005538
mcmv-miR-m88-1-3p	5'-CAGAAGUCGAUGUCGGGGUCU-3'	MIMAT0005552
U6 F	5'-GCTTCGGCAGCACATATACTAAAAT-3'	-
U6 R	5'-CGCTTCACGAATTTGCGTGTCAT-3'	-

Supplementary Table 3: Characteristics of differentially expressed miRNAs in mice induced by MCMV infection

Upregulated miRNAs	log2(FC)	P	Downregulated miRNAs	log2(FC)	P
mmu-miR-211-5p	4.81	<0.001	mmu-miR-7658-3p	-4.32	<0.001
mmu-miR-379-5p	4.00	0.001	9_21079	-4.00	<0.001
mmu-miR-122-5p	3.92	<0.001	11_25868	-3.32	<0.001
mmu-miR-204-5p	3.81	<0.001	mmu-miR-7687-3p	-3.22	<0.001
mmu-miR-183-5p	3.20	<0.001	mmu-miR-486b-3p	-3.09	<0.001
2_3620	3.00	0.005	mmu-miR-8112	-3.00	0.015
mmu-miR-96-5p	2.96	<0.001	15_33426	-2.95	<0.001
mmu-miR-143-3p	2.82	<0.001	mmu-miR-1306-5p	-2.91	<0.001
mmu-miR-434-5p	2.81	0.031	1_2396	-2.74	0.001
17_35459	2.74	0.003	6_12883	-2.59	0.019
16_34132_star	2.70	0.002	8_18027	-2.58	0.017
2_3619	2.66	0.002	7_15813	-2.49	0.013
mmu-miR-133b-3p	2.58	0.050	7_14875	-2.46	0.01
mmu-miR-182-5p	2.57	0.002	2_5312	-2.45	0.003
mmu-miR-382-5p	2.54	0.004	9_20785	-2.38	0.006
mmu-miR-100-5p	2.52	0.007	7_15814	-2.35	0.008
mmu-miR-184-3p	2.47	0.001	mmu-miR-1929-3p	-2.32	0.017
17_35460	2.39	0.005	mmu-miR-5126	-2.28	0.008
mmu-miR-411-5p	2.32	0.020	10_22165_star	-2.19	0.019
mmu-miR-1a-3p	2.31	0.004	7_14962	-2.13	0.019
mmu-miR-203-3p	2.30	0.006	mmu-miR-486a-3p	-2.11	0.032
16_34879	2.17	0.012	11_26101	-2.09	0.014
mmu-miR-429-3p	2.16	0.009	mmu-miR-3102-3p	-2.00	0.030
mmu-miR-3473c	2.07	0.005	mmu-miR-7052-3p	-2.00	0.046
mmu-miR-145a-5p	2.01	0.010	1_639	-1.82	0.037
mmu-miR-145a-3p	2.00	0.035	15_33614	-1.75	0.041
mmu-miR-200b-3p	1.90	0.016	mmu-miR-328-3p	-1.66	0.018
mmu-miR-3473e	1.87	0.015			
mmu-miR-199b-3p	1.83	0.026			
mmu-miR-709	1.81	0.042			
mmu-miR-206-3p	1.80	0.015			
mmu-miR-199a-3p	1.79	0.032			
11_25514	1.78	0.016			
11_25831	1.77	0.039			
mmu-miR-9-5p	1.74	0.040			
5_10470	1.74	0.049			
X_40860	1.70	0.026			
mmu-miR-148a-3p	1.70	0.034			
mmu-miR-200a-3p	1.60	0.041			

mmu-miR-133a-3p	1.60	0.031
mmu-let-7f-5p	1.55	0.047
mmu-miR-3473b	1.54	0.038
mmu-let-7i-5p	1.42	0.040
mmu-let-7a-5p	1.39	0.032
1_335	Inf	0.016
10_21727_star	Inf	0.045
10_22793	Inf	0.021
10_23162	Inf	0.042
11_24008	Inf	0.045
11_24173	Inf	0.015
11_25908	Inf	0.045
11_26241_star	Inf	0.046
14_30406	Inf	0.006
14_30821	Inf	0.027
14_30846	Inf	0.003
14_30905	Inf	0.010
14_31532	Inf	0.045
14_31674	Inf	0.028
15_33456	Inf	0.035
16_34299	Inf	0.006
19_39006	Inf	0.021
2_3169	Inf	0.002
2_4983_star	Inf	0.042
2_5181	Inf	0.027
2_5474_star	Inf	<0.001
3_6204	Inf	0.027
3_6376	Inf	0.027
4_7885	Inf	0.029
4_8698	Inf	0.035
4_9362	Inf	0.041
5_10623	Inf	0.035
5_10674	Inf	0.011
6_13582	Inf	0.021
6_13591	Inf	0.050
6_14044	Inf	0.003
7_15083_star	Inf	0.048
7_15376	Inf	0.034
8_18816	Inf	0.045
9_20233	Inf	0.027
9_21014	Inf	0.045
mmu-miR-124-3p	Inf	<0.001
mmu-miR-1895	Inf	0.045
mmu-miR-216a-5p	Inf	<0.001

mmu-miR-217-5p	Inf	<0.001
mmu-miR-3087-5p	Inf	0.024
mmu-miR-6959-3p	Inf	0.001
mmu-miR-6968-3p	Inf	0.008
mmu-miR-7071-3p	Inf	0.016
mmu-miR-7072-3p	Inf	0.025
mcmv-miR-m01-4-5p		<0.005
mcmv-miR-m88-1-3p		<0.005

Supplementary Table 4: Characteristics of differentially expressed miRNAs in mice induced by MCMV infection

Upregulated miRNAs	log2(FC)	*P*	Downregulated miRNAs	log2(FC)	*P*
mmu-miR-211-5p	4.81	<0.001	mmu-miR-328-3p	-1.66	0.018
mmu-miR-379-5p	4.00	0.001	mmu-miR-3102-3p	-2.00	0.030
mmu-miR-122-5p	3.92	<0.001	mmu-miR-7052-3p	-2.00	0.046
mmu-miR-204-5p	3.81	<0.001	mmu-miR-486a-3p	-2.11	0.032
mmu-miR-183-5p	3.20	<0.001	mmu-miR-5126	-2.28	0.008
mmu-miR-96-5p	2.96	0.001	mmu-miR-1929-3p	-2.32	0.017
mmu-miR-143-3p	2.82	<0.001	mmu-miR-1306-5p	-2.91	<0.001
mmu-miR-434-5p	2.81	0.032	mmu-miR-8112	-3.00	0.015
mmu-miR-133b-3p	2.58	0.050	mmu-miR-486b-3p	-3.09	0.003
mmu-miR-182-5p	2.57	0.002	mmu-miR-7687-3p	-3.22	0.001
mmu-miR-382-5p	2.54	0.004	mmu-miR-7658-3p	-4.32	<0.001
mmu-miR-100-5p	2.52	0.007			
mmu-miR-184-3p	2.47	0.001			
mmu-miR-411-5p	2.32	0.020			
mmu-miR-1a-3p	2.32	0.004			
mmu-miR-203-3p	2.30	0.006			
mmu-miR-429-3p	2.16	0.009			
mmu-miR-3473c	2.07	0.005			
mmu-miR-145a-5p	2.01	0.010			
mmu-miR-145a-3p	2.00	0.035			
mmu-miR-200b-3p	1.90	0.016			
mmu-miR-3473e	1.87	0.015			
mmu-miR-199b-3p	1.83	0.026			
mmu-miR-709	1.81	0.042			
mmu-miR-206-3p	1.80	0.015			
mmu-miR-199a-3p	1.79	0.032			
mmu-miR-9-5p	1.74	0.040			
mmu-miR-148a-3p	1.67	0.034			
mmu-miR-200a-3p	1.61	0.041			
mmu-miR-133a-3p	1.60	0.031			
mmu-let-7f-5p	1.55	0.047			
mmu-miR-3473b	1.54	0.038			
mmu-let-7i-5p	1.42	0.039			
mmu-let-7a-5p	1.39	0.032			
mcmv-miR-m01-4-5p		<0.005			
mcmv-miR-m88-1-3p		<0.005			

Supplementary Table 5: Characteristics of differentially expressed miRNAs related to hypertension in mice induced by MCMV infection

Upregulated miRNAs	log2(FC)	*P*	Downregulated miRNAs	log2(FC)	*P*
mmu-miR-211-5p	4.81	<0.001	mmu-miR-328-3p	-1.66	0.018
mmu-miR-379-5p	4.00	0.001	mmu-miR-3102-3p	-2.00	0.030
mmu-miR-204-5p	3.81	<0.001	mmu-miR-486a-3p	-2.11	0.032
mmu-miR-183-5p	3.20	<0.001	mmu-miR-1929-3p	-2.32	0.017
mmu-miR-143-3p	2.82	<0.001	mmu-miR-1306-5p	-2.91	<0.001
mmu-miR-133b-3p	2.58	0.050	mmu-miR-486b-3p	-3.09	0.003
mmu-miR-182-5p	2.57	0.002			
mmu-miR-382-5p	2.54	0.004			
mmu-miR-411-5p	2.32	0.020			
mmu-miR-1a-3p	2.32	0.004			
mmu-miR-203-3p	2.30	0.006			
mmu-miR-429-3p	2.16	0.009			
mmu-miR-3473c	2.07	0.005			
mmu-miR-145a-5p	2.01	0.010			
mmu-miR-145a-3p	2.00	0.035			
mmu-miR-200b-3p	1.90	0.016			
mmu-miR-709	1.81	0.042			
mmu-miR-206-3p	1.80	0.015			
mmu-miR-9-5p	1.74	0.040			
mmu-miR-148a-3p	1.67	0.034			
mmu-miR-200a-3p	1.61	0.041			
mmu-miR-133a-3p	1.60	0.031			
mmu-let-7f-5p	1.55	0.047			
mmu-miR-3473b	1.54	0.038			
mmu-let-7i-5p	1.42	0.039			
mmu-let-7a-5p	1.39	0.032			

Supplementary Table 6: Enrichment analysis of hypertension-related target genes

Description	pathwayID	p value	genes	enrich_factor
Thyroid cancer	mmu05216	2.072e-06	KRAS TCF7L2 CCND1 RET TRP53	16.66
Phototransduction	mmu04744	3.277e-05	SLC24A1 GRK1 CNGB1 RHO	13.79
Endometrial cancer	mmu05213	4.575e-05	KRAS TCF7L2 CCND1 PTEN TRP53	9.61
Dorso-ventral axis formation	mmu04320	3.261e-03	KRAS NOTCH2	9.09
Aldosterone-regulated sodium reabsorption	mmu04960	2.303e-04	KRAS IRS1 ATP1A2 NEDD4L	9.09
Asthma	mmu05310	4.168e-03	IL10 IL5	8.33
Amyotrophic lateral sclerosis (ALS)	mmu05014	6.913e-04	TNFRSF1B APAF1 MAPK14 TRP53	7.14
Bladder cancer	mmu05219	2.289e-03	KRAS CCND1 TRP53	6.97
Intestinal immune network for IgA production	mmu04672	2.700e-03	IL10 ICOSL IL5	6.66
Prostate cancer	mmu05215	1.192e-04	KRAS TCF7L2 FGFR2 CCND1 PTEN TRP53	6.66
T cell receptor signaling pathway	mmu04660	6.776e-05	IL10 KRAS VAV2 VAV3 IFNG IL5 MAPK14	6.25
Fc epsilon RI signaling pathway	mmu04664	4.681e-04	KRAS VAV2 VAV3 IL5 MAPK14	6.25
African trypanosomiasis	mmu05143	9.282e-03	IL10 IFNG	6.25
Colorectal cancer	mmu05210	1.345e-03	KRAS TCF7L2 CCND1 TRP53	6.15
Leishmaniasis	mmu05140	1.345e-03	IL10 IFNG JAK2 MAPK14	6.15
Glioma	mmu05214	1.439e-03	KRAS CCND1 PTEN TRP53	6.06
p53 signaling pathway	mmu04115	1.864e-03	APAF1 CCND1 PTEN TRP53	5.71
Melanoma	mmu05218	2.108e-03	KRAS CCND1 PTEN TRP53	5.55
Basal cell carcinoma	mmu05217	5.541e-03	TCF7L2 WNT4 TRP53	5.45
Non-small cell lung	mmu05223	5.541e-03	KRAS CCND1	5.45

cancer			TRP53	
Tyrosine metabolism	mmu00350	1.380e-02	DDC COMT	5.40
Allograft rejection	mmu05330	5.905e-03	IL10 IFNG IL5	5.36
Acute myeloid leukemia	mmu05221	6.286e-03	KRAS TCF7L2 CCND1	5.26
Vascular smooth muscle contraction	mmu04270	8.527e-04	EDNRA ADORA2B PRKG1 ARHGEF1 ARHGEF12 ROCK2	4.80
TGF-beta signaling pathway	mmu04350	4.311e-03	SMAD9 IFNG ACVR1 ROCK2	4.70
Small cell lung cancer	mmu05222	4.759e-03	APAF1 CCND1 PTEN TRP53	4.60
Regulation of actin cytoskeleton	mmu04810	6.759e-05	F2R KRAS CSK VAV2 VAV3 FGFR2 ARHGEF1 ARHGEF12 ROCK2 ITGA11	4.59
Adipocytokine signaling pathway	mmu04920	1.162e-02	TNFRSF1B IRS1 JAK2	4.41
Malaria	mmu05144	2.618e-02	IL10 IFNG	4.25
Pancreatic cancer	mmu05212	1.347e-02	KRAS CCND1 TRP53	4.22
Leukocyte transendothelial migration	mmu04670	3.697e-03	VAV2 VAV3 RHOH MAPK14 ROCK2	4.17
Chronic myeloid leukemia	mmu05220	1.550e-02	KRAS CCND1 TRP53	4.05
Melanogenesis	mmu04916	8.879e-03	EDN1 KRAS TCF7L2 WNT4	3.96
B cell receptor signaling pathway	mmu04662	1.696e-02	KRAS VAV2 VAV3	3.95
Jak-STAT signaling pathway	mmu04630	2.698e-03	IL10 SPRY2 IFNG CCND1 IL5 JAK2	3.92
Wnt signaling pathway	mmu04310	2.798e-03	TCF7L2 CCND1 SOX17 WNT4 ROCK2 TRP53	3.89
Neurotrophin signaling pathway	mmu04722	5.875e-03	KRAS IRS1 CSK MAPK14 TRP53	3.79
Chemokine signaling pathway	mmu04062	2.115e-03	GRK1 KRAS CSK VAV2 VAV3 JAK2 ROCK2	3.70
Focal adhesion	mmu04510	2.993e-03	RELN VAV2 VAV3 CCND1 PTEN ROCK2 ITGA11	3.50

Gap junction	mmu04540	2.762e-02	KRAS PRKG1 MAP3K2	3.41
Cytokine-cytokine receptor interaction	mmu04060	2.935e-03	TNFRSF1B IL10 LTA IFNG ACVR1 IL17RB IL5 TNFSF4	3.25
Natural killer cell mediated cytotoxicity	mmu04650	2.094e-02	KRAS VAV2 VAV3 IFNG	3.20
Type I diabetes mellitus	mmu04940	5.579e-02	LTA IFNG	3.17
Toxoplasmosis	mmu05145	2.297e-02	IL10 IFNG JAK2 MAPK14	3.12
GnRH signaling pathway	mmu04912	4.042e-02	KRAS MAP3K2 MAPK14	3.03
Chagas disease (American trypanosomiasis)	mmu05142	4.308e-02	IL10 IFNG MAPK14	2.97
Autoimmune thyroid disease	mmu05320	7.777e-02	IL10 IL5	2.78
Long-term depression	mmu04730	7.777e-02	KRAS PRKG1	2.78
Arrhythmogenic right ventricular cardiomyopathy (ARVC)	mmu05412	8.317e-02	TCF7L2 ITGA11	2.70
Complement and coagulation cascades	mmu04610	8.875e-02	F2R THBD	2.63
VEGF signaling pathway	mmu04370	8.875e-02	KRAS MAPK14	2.63
Salivary secretion	mmu04970	9.160e-02	PRKG1 ATP1A2	2.60
Pathways in cancer	mmu05200	1.715e-02	KRAS TCF7L2 FGFR2 CCND1 WNT4 PTEN RET TRP53	2.45
Rheumatoid arthritis	mmu05323	1.160e-01	ANGPT1 IFNG	2.35
Cell cycle	mmu04110	8.930e-02	CDKN1C CCND1 TRP53	2.34
ECM-receptor interaction	mmu04512	1.193e-01	RELN ITGA11	2.32
Apoptosis	mmu04210	1.193e-01	APAF1 TRP53	2.32
Progesterone-mediated oocyte maturation	mmu04914	1.259e-01	KRAS MAPK14	2.27
Axon guidance	mmu04360	9.780e-02	KRAS ARHGEF12 ROCK2	2.27
Endocytosis	mmu04144	6.082e-02	GRK1 F2R FGFR2 RET NEDD4L	2.24

Tight junction	mmu04530	1.090e-01	KRAS HCLS1 PTEN	2.19
Hepatitis C	mmu05160	1.090e-01	KRAS MAPK14 TRP53	2.19
Fc gamma R-mediated phagocytosis	mmu04666	1.396e-01	VAV2 VAV3	2.17
Neuroactive ligand-receptor interaction	mmu04080	5.524e-02	EDNRA ADORA2B ADRA2A F2R ADRA2B NR3C1	2.17
MAPK signaling pathway	mmu04010	1.259e-01	KRAS FGFR2 MAP3K2 MAPK14 TRP53	1.86
Amoebiasis	mmu05146	2.344e-01	IL10 IFNG	1.72
Osteoclast differentiation	mmu04380	2.432e-01	IFNG MAPK14	1.69
Calcium signaling pathway	mmu04020	2.247e-01	EDNRA ADORA2B F2R	1.68
Insulin signaling pathway	mmu04910	3.315e-01	KRAS IRS1	1.46
Systemic lupus erythematosus	mmu05322	4.067e-01	IL10 IFNG	1.32
Alzheimer's disease	mmu05010	6.033e-01	APAF1 ERN1	1.06
Staphylococcus aureus infection	mmu05150	1	IL10	1.00
Parkinson's disease	mmu05012	1	APAF1	1.00
Nicotinate and nicotinamide metabolism	mmu00760	1	NMNAT2	1.00
Proximal tubule bicarbonate reclamation	mmu04964	1	ATP1A2	1.00
Biosynthesis of unsaturated fatty acids	mmu01040	1	ELOVL6	1.00
Dilated cardiomyopathy	mmu05414	1	ITGA11	1.00
Renal cell carcinoma	mmu05211	1	KRAS	1.00
Proteasome	mmu03050	1	IFNG	1.00
Arachidonic acid metabolism	mmu00590	1	PTGS1	1.00
Cardiac muscle contraction	mmu04260	1	ATP1A2	1.00
Histidine metabolism	mmu00340	1	DDC	1.00
Hematopoietic cell lineage	mmu04640	1	IL5	1.00
NOD-like receptor	mmu04621	1	MAPK14	1.00

signaling pathway				
Alanine, aspartate and glutamate metabolism	mmu00250	1	GPT2	1.00
Arginine and proline metabolism	mmu00330	1	OTC	1.00
Toll-like receptor signaling pathway	mmu04620	1	MAPK14	1.00
Tryptophan metabolism	mmu00380	1	DDC	1.00
Phenylalanine metabolism	mmu00360	1	DDC	1.00
Graft-versus-host disease	mmu05332	1	IFNG	1.00
Hedgehog signaling pathway	mmu04340	1	WNT4	1.00
Protein processing in endoplasmic reticulum	mmu04141	1	ERN1	1.00
Adherens junction	mmu04520	1	TCF7L2	1.00
Gastric acid secretion	mmu04971	1	ATP1A2	1.00
Huntington's disease	mmu05016	6.574e-01	APAF1 TRP53	1.00
Selenocompound metabolism	mmu00450	1	CTH	1.00
Viral myocarditis	mmu05416	1	CCND1	1.00
ErbB signaling pathway	mmu04012	1	KRAS	1.00
Ubiquitin mediated proteolysis	mmu04120	1	NEDD4L	1.00
Cell adhesion molecules (CAMs)	mmu04514	1	ICOSL	1.00
Type II diabetes mellitus	mmu04930	1	IRS1	1.00
Regulation of autophagy	mmu04140	1	IFNG	1.00
Notch signaling pathway	mmu04330	1	NOTCH2	1.00
Pancreatic secretion	mmu04972	1	ATP1A2	1.00
RNA degradation	mmu03018	1	SKI	1.00
Antigen processing and presentation	mmu04612	1	IFNG	1.00
Glycine, serine and threonine metabolism	mmu00260	1	CTH	1.00
Nitrogen metabolism	mmu00910	1	CTH	1.00
Fat digestion and absorption	mmu04975	1	MTTP	1.00

Phosphatidylinositol signaling system	mmu04070	1	PTEN	1.00
Long-term potentiation	mmu04720	1	KRAS	1.00
Protein digestion and absorption	mmu04974	1	ATP1A2	1.00
Bile secretion	mmu04976	1	ATP1A2	1.00
Carbohydrate digestion and absorption	mmu04973	1	ATP1A2	1.00
Hypertrophic cardiomyopathy (HCM)	mmu05410	1	ITGA11	1.00
Bacterial invasion of epithelial cells	mmu05100	1	HCLS1	1.00
Inositol phosphate metabolism	mmu00562	1	PTEN	1.00
Steroid hormone biosynthesis	mmu00140	1	COMT	1.00
Cysteine and methionine metabolism	mmu00270	1	CTH	1.00
Circadian rhythm	mmu04710	1	ARNTL	1.00
ABC transporters	mmu02010	1	ABCC9	1.00
RIG-I-like receptor signaling pathway	mmu04622	1	MAPK14	1.00
Metabolic pathways	mmu01100	1	CTH DDC COMT GPT2 PTGS1 NMNAT2 OTC	0.59
Olfactory transduction	mmu04740	1	CNGB1 PDC PRKG1	0.30

Supplementary Table 7: miRNA-mRNA interaction analysis and regulatory network construction

miRNA	Target gene
mmu-let-7a-5p	Edn1, Pctp, Map3k2
mmu-let-7f-5p	Edn1, Hmga1, Pctp, Map3k2
mmu-let-7i-5p	Edn1, Hmga1, Pctp
mmu-miR-1306-5p	Apaf1, Arhgef12
mmu-miR-133a-3p	Agt, Otc, Ptpro, Myrf
mmu-miR-133b-3p	Agt, Otc, Ptpro, Myrf
mmu-miR-143-3p	Clcn3, Kras, Asb4, Tardbp
mmu-miR-145a-5p	Ddc, Irs1, Mttp, Il17rb, Kcnk6, Nedd4l, Myrf, Adam1a
mmu-miR-148a-3p	Kcnj8, Pten, Cth, Itga11
mmu-miR-182-5p	Acvr1, Adra2a, Otc, Phb, Rgs2, Wnt4
mmu-miR-183-5p	Clcn3, Irs1, Nrp2, Tcf7l2, Spry2
mmu-miR-1a-3p	Clcn3, Edn1, Epcam, Rgs2, Smarca4, Sox17, Corin
mmu-miR-200a-3p	Nr3c1, Pten, Sox17, Tfap2b, Thbd, Corin
mmu-miR-200b-3p	F2r, Nr3c1, Il10, Ntf3, Pkd1, Reln
mmu-miR-203-3p	Adra2b, Arntl, Fkbp1b, Nr3c1, Prkg1, Nedd4l, Atp1a2, Tardbp
mmu-miR-204-5p	Comt, Nr3c1, Il10, Ddr2, Prkg1, Ski, Wnt4, Nedd4l, Elovl6, Dicer1
mmu-miR-206-3p	Clcn3, Edn1, Epcam, Rgs2, Sox17, Corin
mmu-miR-211-5p	Comt, Nr3c1, Il10, Ddr2, Prkg1, Ski, Wnt4, Elovl6, Dicer1
mmu-miR-328-3p	Nr3c1, Tcf7l2, Trp53
mmu-miR-3473b	Pten, Vav3
mmu-miR-3473c	Irs1, Lta, Ret, Rock2, Abcc9, Nol3, Slc43a2
mmu-miR-382-5p	Ifng, Kras, Ptgs1, Arhgef12, Rhoh
mmu-miR-429-3p	F2r, Nr3c1, Il10, Ntf3, Pkd1, Reln
mmu-miR-9-5p	Cdkn1c, Erg, Notch2, Tnfsf4, Vav3
mmu-miR-124-3p	Angpt1, Rock2, Map3k2, Mapk14, Fstl3, Gpt2, Myrf, Nr3c1, Arhgef1, Ern1
mmu-miR-145a-3p	Acvrl1, Ccnd1, Fgfr2, Mnat1, Slc43a2, Il5, Irs1
mmu-miR-1895	Pdc
mmu-miR-1929-3p	Ednra, Htt, Smad9
mmu-miR-216a-5p	Csk, F2r, Ddr2, Tdrd7, Gpt2, Jak2
mmu-miR-217-5p	Kras, Tdrd7
mmu-miR-3087-5p	Ednra, Htt, Stim1, Slc2a9, Rho, Slc24a1, Sh3pxd2b, Add2, Wt1
mmu-miR-3102-3p	Nmnat2, Nyx, Pcdh15, F2r, Nol3
mmu-miR-379-5p	Edn1
mmu-miR-411-5p	Icosl, Myrf, Angpt1
mmu-miR-709	Hcls1, Grk1, Nr5a1, Corin, Slc43a2, Cngb1, Adora2b
mmu-miR-486b-3p	Hmga1, Vav2, Tnfrsf1b, Pkd1, Itga11
mmu-miR-486a-3p	Hmga1, Vav2, Tnfrsf1b, Pkd1

Supplementary Table 8: Differential miRNAs regulate target genes' GO enrichment results (Top 20)

GO_ID	Term	GO_namespace	gene_num
GO:0005623	cell	CC	1146
GO:0044464	cell part	CC	1145
GO:0009987	cellular process	BP	1071
GO:0005488	binding	MF	979
GO:0043226	organelle	CC	951
GO:0044699	single-organism process	BP	923
GO:0065007	biological regulation	BP	837
GO:0008152	metabolic process	BP	820
GO:0050789	regulation of biological process	BP	793
GO:0016020	membrane	CC	643
GO:0044422	organelle part	CC	629
GO:0050896	response to stimulus	BP	581
GO:0032501	multicellular organismal process	BP	510
GO:0071840	cellular component organization or biogenesis	BP	484
GO:0044425	membrane part	CC	471
GO:0048518	positive regulation of biological process	BP	461
GO:0032502	developmental process	BP	461
GO:0051179	localization	BP	453
GO:0023052	signaling	BP	444
GO:0048519	negative regulation of biological process	BP	406

Supplementary Table 9: KEGG Pathway enrichment results of differential miRNAs regulating target genes (Top 20)

ID	Description	GeneRatio	*p* value	*p*.adjust
mmu04010	MAPK signaling pathway	34/521	<0.001	0.1180
mmu04330	Notch signaling pathway	10/521	0.001	0.1188
mmu04721	Synaptic vesicle cycle	11/521	0.001	0.1497
mmu04668	TNF signaling pathway	15/521	0.004	0.2528
mmu04210	Apoptosis	17/521	0.005	0.2528
mmu04724	Glutamatergic synapse	15/521	0.005	0.2528
mmu04120	Ubiquitin mediated proteolysis	17/521	0.006	0.2676
mmu05031	Amphetamine addiction	10/521	0.010	0.3195
mmu05221	Acute myeloid leukemia	10/521	0.011	0.3195
mmu04014	Ras signaling pathway	24/521	0.012	0.3195
mmu04024	cAMP signaling pathway	21/521	0.012	0.3195
mmu04215	Apoptosis - multiple species	6/521	0.013	0.3195
mmu04360	Axon guidance	19/521	0.014	0.3195
mmu05231	Choline metabolism in cancer	12/521	0.021	0.4242
mmu04392	Hippo signaling pathway - multiple species	5/521	0.021	0.4242
mmu04211	Longevity regulating pathway	11/521	0.025	0.4573
mmu00250	Alanine, aspartate and glutamate metabolism	6/521	0.027	0.4573
mmu00220	Arginine biosynthesis	4/521	0.028	0.4573
mmu05030	Cocaine addiction	7/521	0.029	0.4573
mmu00310	Lysine degradation	8/521	0.030	0.4573
